# Detecting cardiac contractile activity in the early mouse embryo using multiple modalities

**DOI:** 10.3389/fphys.2014.00508

**Published:** 2015-01-07

**Authors:** Chiann-Mun Chen, António M. A. Miranda, Gil Bub, Shankar Srinivas

**Affiliations:** ^1^Department of Physiology Anatomy and Genetics, University of OxfordOxford, UK; ^2^Wellcome TrustLondon, UK

**Keywords:** mouse embryo, cardiac development, time lapse imaging, atomic force microscopy, contraction mapping, calcium transients

## Abstract

The heart is one of the first organs to develop during mammalian embryogenesis. In the mouse, it starts to form shortly after gastrulation, and is derived primarily from embryonic mesoderm. The embryonic heart is unique in having to perform a mechanical contractile function while undergoing complex morphogenetic remodeling. Approaches to imaging the morphogenesis and contractile activity of the developing heart are important in understanding not only how this remodeling is controlled but also the origin of congenital heart defects (CHDs). Here, we describe approaches for visualizing contractile activity in the developing mouse embryo, using brightfield time lapse microscopy and confocal microscopy of calcium transients. We describe an algorithm for enhancing this image data and quantifying contractile activity from it. Finally we describe how atomic force microscopy can be used to record contractile activity prior to it being microscopically visible.

## Introduction

The heart is one of the first organs to develop during mammalian embryogenesis. In the mouse, it starts to form shortly after gastrulation, roughly at embryonic day 7.5 (E7.5). It is derived primarily from mesodermal cells that pass through the early primitive streak, migrating rostrally, and laterally to occupy a crescent shaped region called the “cardiac crescent.” Once there, these cells start to differentiate into cardiomyocytes. Concomitant with this, embryonic folding causes the cardiac crescent to converge on the midline between E7.5 and E8.0, leading to the formation of a “linear heart tube” with the venous pole (inflow) at the caudal end and the arterial pole (outflow) at the rostral end. This structure then undergoes a characteristic dextral looping at around E8.5 that leads to the venous pole being positioned more rostrally. Following this, over a period of days it undergoes extensive remodeling and septation, resulting in the formation of the four chambers, valves, etc. (reviewed in Harvey, 2002; Buckingham et al., 2005; Barnett et al., 2012; Kelly, 2012).

The heart is unique in having to perform a mechanical contractile function while undergoing this complex morphogenetic remodeling. There is evidence that the hemodynamic forces produced by the contractile activity are important for the proper development of the heart in zebrafish (Peralta et al., 2013; Plavicki et al., 2014) and chick (Midgett and Rugonyi, 2014) and embryonic vasculature in mouse (Lucitti et al., 2007). Approaches to imaging the morphogenesis and contractile activity of the developing heart are important in understanding not only how this process is controlled but also the origin of congenital heart defects (CHDs), which are the most common form of birth defect worldwide, occurring in approximately 4 per 1000 live births (Marelli et al., 2007). It is also important in giving us insights into how embryonic pathways can be activated in situations of heart injury to promote cardiomyocyte differentiation for tissue repair.

Ca^2+^ is crucial for several physiological and morphological processes, being involved in many signaling cascades and binding to structural proteins, altering their function. One of the most studied roles of Ca^2+^ is in heart physiology, where the regulation of the cytosolic concentration of calcium by ion channels on the cell membrane and in intracellular stores needs to be fine-tuned to allow the heart to contract properly. Many heart diseases arise as a consequence of disruptions in this process and are often fatal (Kawashiri et al., 2014). The role of Ca^2+^ in the embryonic mouse heart is much less well-understood. Here we report various approaches for the visualization of heart development, contractile activity, and Ca^2+^ transients in the mouse embryo.

## Methods

### Mouse strains, husbandry, and embryo collection

The Hex-GFP line genetically modified mice were maintained on a mixed C57Bl/6 CBA/J background. We used homozygous Hex-GFP studs to cross with CD1 females (Charles River) to generate embryos for the brightfield experiments. Wild type C57Bl/6 studs were crossed with CD1 females to generate embryos for Ca2^+^ imaging. All mice were maintained on a 12 h light, 12 h dark cycle. Noon on the day of finding a vaginal plug was designated embryonic day 0.5 (E0.5).

### Brightfield imaging

To immobilize embryos for the duration of imaging, we prepared glass bottom dishes (MatTek Corporation; P35G-1.0-14-C) prior to the addition of medium by placing paired spots of vacuum grease (Dow Corning high vacuum grease) to serve as anchors for an arch of vacuum grease. We filled a 1 cc syringe with vacuum grease and adapted it with a 20 μl pipette tip in order to be able to dispense controlled amounts of vacuum grease at precise locations. Sufficient medium was dispensed into the dish to fill the central depression and then arches of vacuum grease made using the existing anchors. We then placed the embryo under the arch in the desired orientation and patted down the vacuum grease to hold it firmly. Finally, the culture medium was overlaid with pre-equilibrated mineral oil (Sigma-Aldrich; M8410) to prevent evaporation (Supplementary Figure 1A). Alternately, we made two lines of vacuum grease spanning the diameter of the cover-slip depression and used arches of vacuum grease spanning these two lines to immobilize several embryos in a line (Supplementary Figure 1B).

Embryos were imaged on a Spinning disk confocal (Improvision) built around a Zeiss Axiovert inverted microscope. The spinning disk head was a Yokogawa CSU10 and the detector was a Hammamatsu emCCD camera with 512 × 512 detector. The microscope had an environmental control chamber (Solent Scientific) with temperature control and humidified 5% CO_2_. Time-lapse experiments were controlled using the Volocity program (Improvision – Perkin Elmer). Embryos were imaged using brightfield illumination, and 488 nm excitation for Hex-GFP. Ten z-levels were captured at each time point, with an 8 min time-lapse between time points. Exposure time per section was 100 ms for the brightfield channel and 400 ms for the GFP channel.

### Image processing

The *temporal normalization* image filter scales each pixel so that:

$$p_{t}(x,y)=\frac{p_{t}(x,y)-p_{min}(x,y)}{p_{max}(x,y)-p_{min}(x,y)}$$

where *p_t_*(*x, y*) is the intensity of a pixel located at position *x, y* for frame *t, p_min_*(*x, y*) is the minimum intensity for that pixel location, and *p_max_*(*x, y*) is the maximum intensity for that pixel location for all frames in an image sequence.

The *absolute difference* image filter is applied to image series to visually enhance tissue motion: *p_t_*(*x, y*) = |*p_t_*(*x, y*) − *p*_*t* − *N*_(*x, y*)| where *p_t_*(*x, y*) is the intensity of a pixel located at position *x, y* for frame *t*, and *pV*_*t* − *N*_(*x, y*) is the intensity of the same pixel N frames prior. Intensity *vs*. time plots, obtained either from the image sequence directly or from the absolute difference filtered image sequences can be processed using standard spectral methods (e.g., discrete Fourier transform) or by a simple algorithms that measures the time between peaks and their variance. All image processing code is custom written in Java and available on request.

### Imaging calcium transients

We dissected embryos in CO_2_ independent media M2 (Sigma) and exposed the cardiac tissue by removing the overlaying endoderm and pericardial sac. We then incubated embryos in 5 μM Rhod-2/AM (Life Technologies, R-1244) in culture media, which is a 1:1 mix of CMRL (Invitrogen) and KnockOut Serum (Gibco), at 37°C in 5% CO_2_. Pluoronic acid at a final concentration of 0.02% can be used to permeabilize the embryos, but we found it is unnecessary if using an AM dye. We prepared in advance MatTek dishes to receive the stained embryos as for brightfield imaging. After staining embryos for 15 min, we transferred them into the previously prepared MatTek dish and positioned them with the cardiac tissue facing the coverslip of the dish. We immobilized embryos with an arch of vacuum grease over each individual embryo and then imaged them using a Zeiss 710 confocal microscope fitted with an environmental chamber to maintain the embryo at 37°C at 5% CO_2_. Embryos were imaged with a 20× 0.5 NA objective using a 564 nm laser for excitation. We captured a single optical section every 97 ms (~10 frames per second). Images were captured at 256 × 256 pixel dimensions, with a 2× line step and no averaging to increase the scan speed.

To remove background non-dynamic signal from the Rhod-2 images we used Fiji. We duplicated a single frame from the Rhod-2 channel where the embryo was in the resting phase of the transient (time between two consecutive transients). Then, using the “Image Calculator” utility (under Process > Image Calculator), we subtracted this duplicated frame from all the frames within the time-series. This will create a new channel (“ΔRhod-2”) that can be merged with the Brightfield channel for visualization of the calcium transient within the context of the embryo. For optimal visualization, the Spectrum LUT can be applied to the newly created channel to visualize intensity changes in the transient.

### Atomic force microscopy

For AFM measurements, we used a JPK NanoWizard 3, installed on a Zeiss AxioObserver D1. The AFM cantilever (Arrow TL2 probes from NanoWorld) was prepared by attaching 11.2 μm diameter latex beads attached with two component epoxy as per manufacturer's recommendations. The cantilevers were calibrated using the thermal tune method. Embryos were dissected in M2 medium (Sigma) at room temperature, transferred to a fresh dish of M2 at room temperature and immobilized with hand pulled glass capillaries of 15 to 20 μm diameter under a dissection microscope. They were then transferred to the AFM for measurement. The region of the heart to probe was determined visually by monitoring the optical image of the embryo through the Zeiss AxioObserver. The force spectroscopy curves we took had a significant dwell time on the surface, during which the “constant height mode” we used to gather measurements. Force measurements data were processed in Microsoft Excel to generate plots of changes in force over time.

### Statistical analysis

For the statistical analysis of change in beat rate of cultured embryos, we performed a One-Way ANOVA followed by a *post-hoc* Tukey HSD test with a 95% family-wise confidence level to calculate the difference between the four groups (2, 4, 8, and 13 h). The null hypothesis was rejected for *p*-values < 0.05. All tests were performed using R software.

## Results and discussion

### Brightfield imaging of developing cardiac crescent stage embryos

To image contractile activity in the developing cardiac primordium, we adapted static culture approaches that have been previously described (Jones et al., 2002; Nowotschin et al., 2010; Srinivas, 2010) for visualizing contractile activity at high spatial (micron scale) and temporal (up to 10 frames per second) resolution.

Gastrulation stage mouse embryos are amenable to time-lapse imaging as they can be maintained in static culture for periods in excess of 12 h. However, imaging mouse embryos poses several unique challenges. Mouse embryos are relatively delicate compared to those of other model systems such as zebrafish or Drosophila, and are much less tolerant to variations in culture conditions (media composition, temperature, humidity, pH, etc.). Cardiac tissue in the early embryo is poorly striated and, therefore, intrinsically low contrast relative to adult tissue, making it difficult to visualize. Furthermore, the embryo undergoes very rapid and extensive growth at this stage, which causes it to change its position and roll out of the field of view, posing a major hurdle to extended imaging. This type of movement is all the more problematic in imaging experiments of a particular region of the embryo such as the forming heart, since the rolling of the embryo can, over time, angle this region out of view or shift it out of the focal plane.

Any method used to immobilize the embryo risks changing its developmental profile and therefore has to be used with care. We tried several approaches to restrict the movement of the embryo including embedding the embryo in agarose or a fibrin clot. Such approaches were not suitable as they perturbed development, either mechanically, by preventing it from expanding to the normal extent, or chemically. We also immobilized embryos between two parallel hand-pulled glass filaments fixed to the dish with vacuum grease. This was effective in restricting the embryo while allowing sufficient flexibility for repositioning it to present the desired region for imaging. We however found that over the course of the overnight culture, the embryo grew extensively, leading to it being dislodged from between the glass filaments or alternately, becoming squeezed and distorted between the glass filaments.

We therefore developed an approach to immobilize the embryo under an “arch” of vacuum grease (Supplementary Figure 1A). Such vacuum grease is composed of chemically inert parrafins and does not perturb development. We first spotted vacuum grease onto a glass bottom dish, filled it with culture medium and then extruded thin cables of vacuum grease in arches within the medium. The spots of vacuum grease applied before adding the medium help anchor the arches, which otherwise have a tendency to float away to the surface of the medium. Dissected embryos could then be slipped under these arches, and the vacuum grease gently patted down to restrict the movement of the embryo. These arches were sufficient to restrict the movement of the embryo but flexible enough to deform with its growth. A variation of this approach, suitable for imaging several embryos, is to lay down two parallel “tracks” of vacuum grease along the bottom of a glass-bottomed dish (Supplementary Figure 1B). The two lines of vacuum grease are separated by a space roughly equivalent to the diameter of an embryo. We then place several strands of vacuum grease perpendicularly on top of the two “rails,” to form a ladder like pattern. Embryos can now be placed between the two rails and beneath a “rung,” which can be patted down to restrict the movement of the sample. This latter approach allows us to easily image several embryos along a straight line.

**Figure 1:**
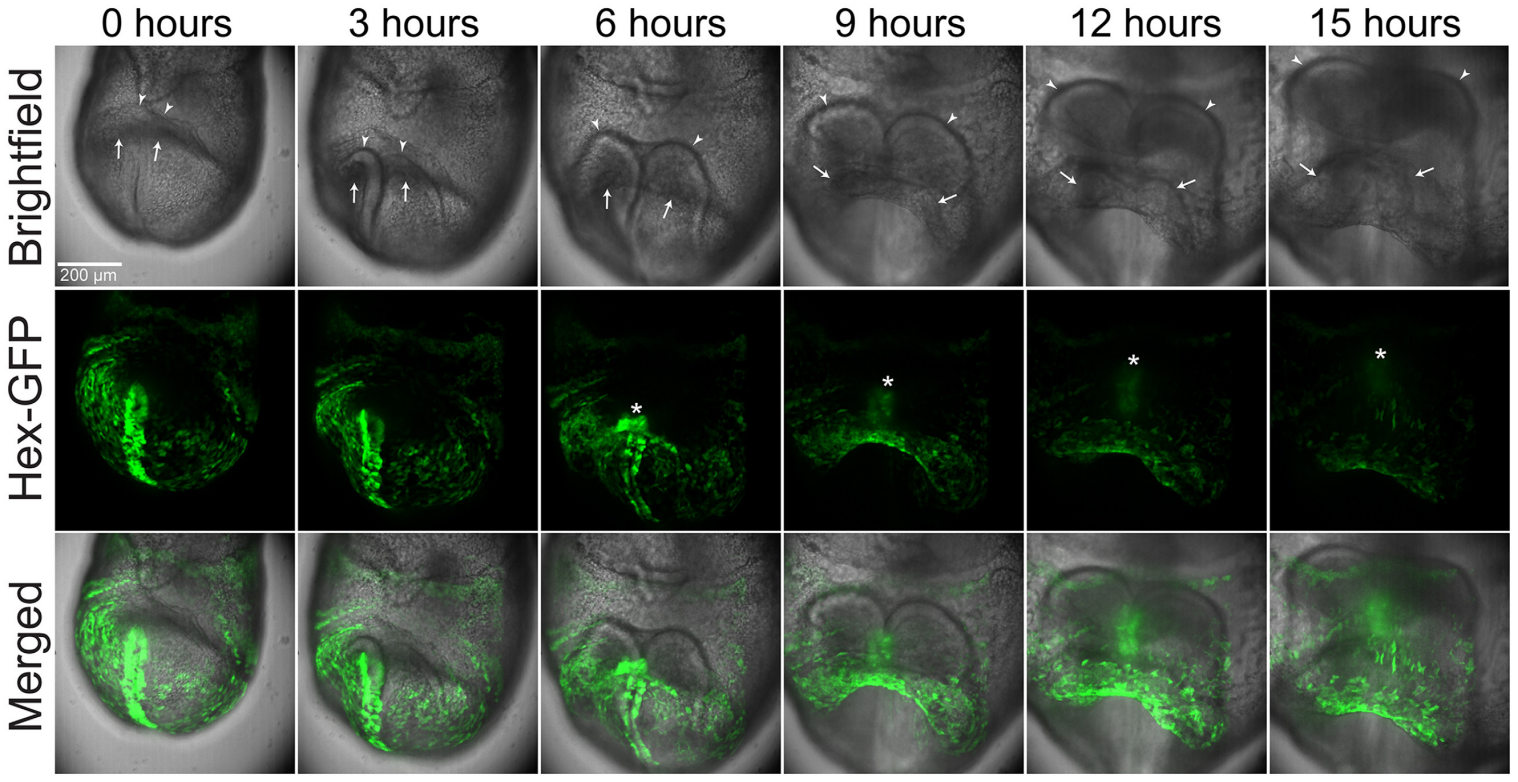
**Stills from a time-lapse sequence of a late E7.5 imaged by brightfield and GFP fluorescence microscopy**. The egg cylinder is orientated with the distal end toward the bottom, the proximal end toward the top, and the forming rostral aspects facing the viewer. Hex-GFP labels cells of the endoderm. The arrow point to the forming heart, the arrowheads to the neural folds and the asterisk to the foregut diverticulum. Image volumes were captured at 8 min intervals. The scale bar represents 200 microns. Also see Supplementary Movie 1.

To image the development of the linear heart tube from the cardiac crescent, we dissected E7.75 Hex-GFP transgenic mouse embryos in M2, taking care to not damage the yolk sac and to leave the ectoplacental cone intact. The Hex-GFP transgene labels the visceral and definitive endoderm, allowing us to more readily visualize morphogenetic movements (Rodriguez et al., 2001). We then transferred the embryos into a glass-bottomed dish with culture media and positioned them under the vacuum grease restraints. We cultured embryos in an incubated spinning disk confocal microscope for up to 20 h while imaging them periodically (see Methods for details). Embryos developed well from early cardiac crescent to late linear heart tube stages, over a period of ~15 h. Time-lapse recordings show the rostral and lateral folding movements that help shape the heart into a linear heart. In contrast to the chick, where the two lateral limbs of cardiac crescent swing down and meet in the midline to form the linear heart tube, in the mouse we observe more of a convergence toward the midline, particularly in the more rostral aspect of the crescent, so that the resulting “linear” heart tube is in fact the somewhat spherical shape characteristic of mouse embryonic hearts (Figure 1, white arrows and Supplementary Movie 1). We are also able to clearly visualize the rostral folding movements (Madabhushi and Lacy, 2011) that are important in bringing the forming heart to its more caudal position with respect to the forming brain. Positioning the embryo in this manner also allowed us to record the formation of the foregut diverticulum (Figure 1 and Supplementary Movie 1).

**Figure 2:**
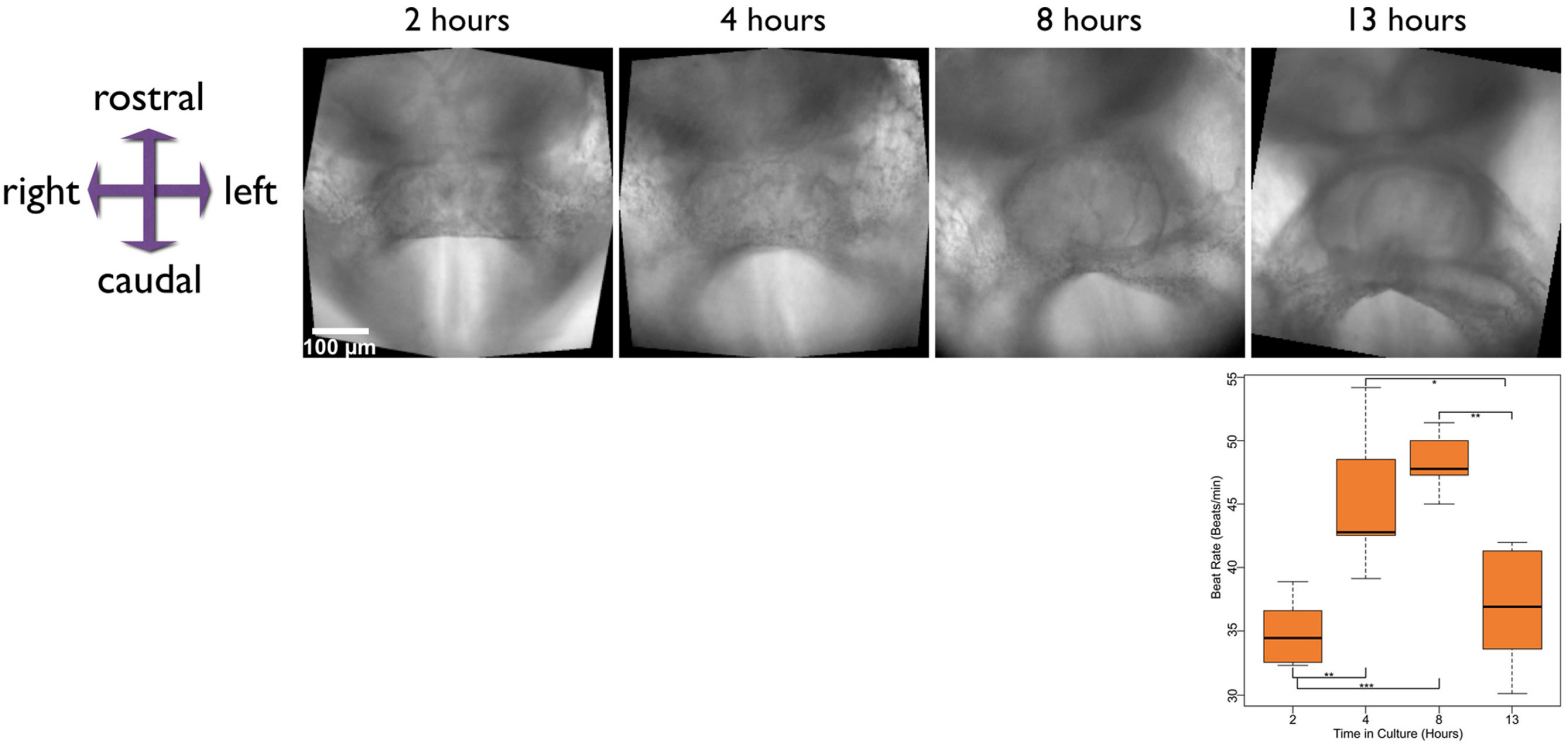
**Stills from time-lapse movies taken at various stages in the development of a late E7.5 embryo, developing from the cardiac crescent to linear heart tube stages**. The time above each image is measured from the start of culture. Time-lapse movies were captured at each of these stages to capture the contractile activity of the developing heart. The chart plots the beat rate of hearts from five different embryos, at the stages shown. The scale bar represents 100 microns. ^*^ ≤ 0.05, ^**^ ≤ 0.01, and ^***^ ≤ 0.001. Also see Supplementary Movie 2.

In addition to imaging morphogenesis of the heart in the developing embryo, we also modified the imaging protocol to visualize contractile activity more clearly. For this, we positioned embryos at the bottom of a glass bottomed dish as above, and then at regular intervals of 10 min, took high frame rate 10 s movies of the developing heart, to record the emergence of contractile activity over time. We cultured embryos for over 15 h and recorded movies of contractile activity at various stages (Figure 2 and Supplementary Movie 2). We also quantified beat rate in five cultured embryos as they progressed through different stages of development (see Section below on enhancing and quantifying contractile activity) and found the rate increased for approximately the first 8 h in culture, after which it leveled off and then started to drop. The drop in beat rate is likely due to a change in pH of the culture medium over the duration of the culture. Using larger culture chambers with a larger volume of medium, or perfusion of fresh medium into the culture chamber might be future refinements to the culture set up that will overcome this potential limitation.

### Imaging calcium transients in the developing heart

While the regulation and function of calcium in cardiac contractions has been extensively studied in adult tissues, relatively little is known about the role of calcium physiology during embryonic development. One of the physiological manifestations of a functioning calcium handling machinery is the presence of calcium transients. Therefore, to further understand the importance of calcium physiology during development, it is important to be able to characterize these calcium transients, investigate their patterns and determine how they are affected when different ion channels are perturbed. Having detected contractile activity by brightfield microscopy, we next developed an approach to image calcium transients in the E8.0 mouse embryo.

**Figure 3:**
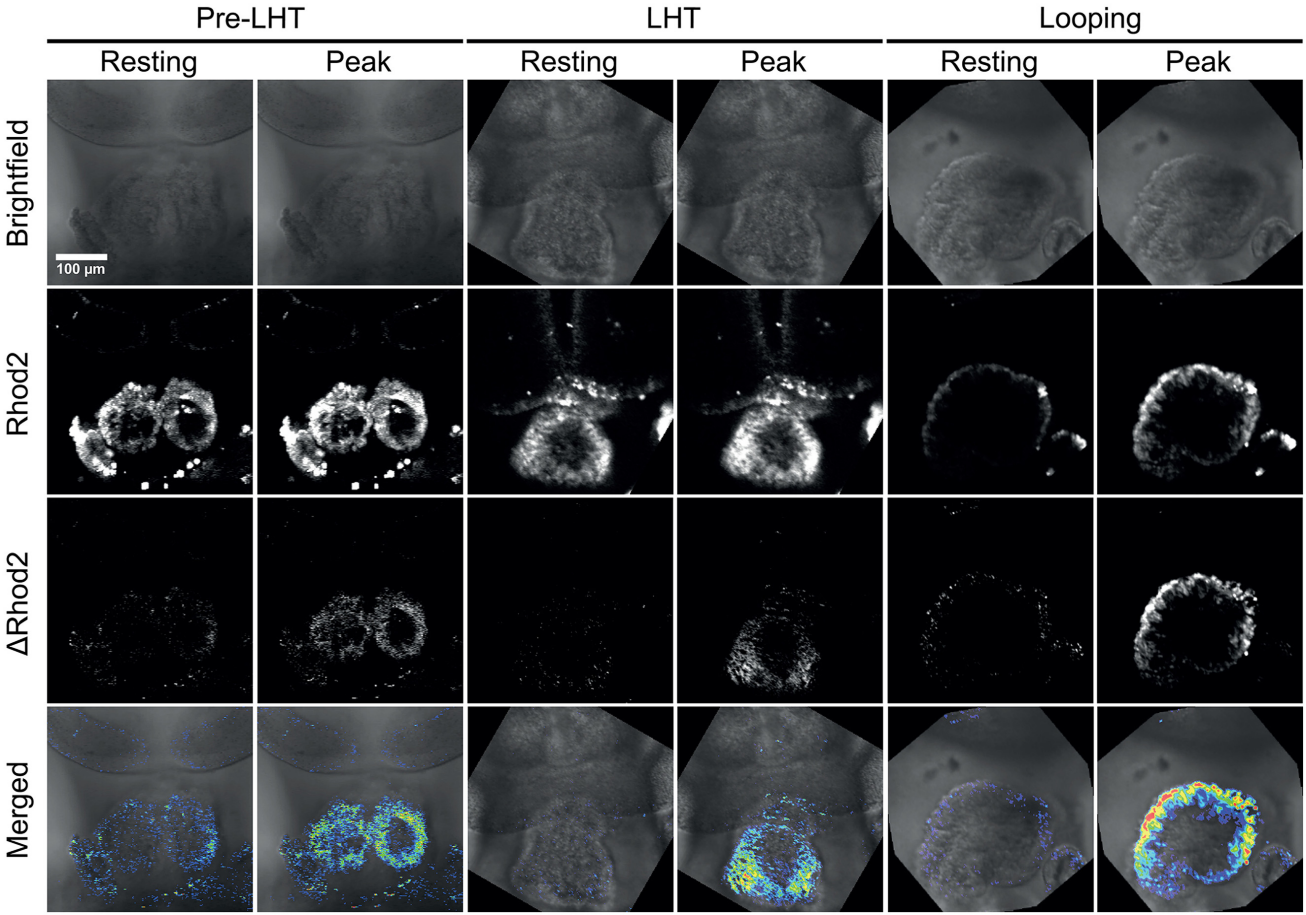
**Stills from time-lapse sequences of Ca^2+^ transients in three stages of early heart development**. The stages shown are pre-linear heart tube, linear heart tube, and early looping heart tube. The pericardial sac was removed to allow entry of the Ca^2+^ sensitive dye Rhod-2. The scale bar represents 100 microns. Also see Supplementary Movie 3.

We isolated embryos at several stages of development and exposed the cardiac tissue by removing the overlaying endoderm and pericardial sac. This is essential to allow the calcium sensitive dye to penetrate the developing myocardium. Next, we incubated embryos in 5 μM calcium dye Rhod-2 for 15 min, and then immobilized the embryos for imaging as for brightfield microscopy. Embryos were imaged with a laser scanning confocal microscope using parameters that allowed for scanning at 10 frames per second. We detected clear calcium transients progressing across the forming linear and looping heart tube, indicating that excitation contraction coupling is likely already in place at this stage of development (Figure 3 and Supplementary Movie 3). In order to visualize the calcium transients more clearly, we performed an image subtraction to remove the baseline fluorescence from each frame of the time-lapse movie, so that the change in intensity of the calcium dye (“ΔRhod-2”) was more clearly visible (Figure 3 and Supplementary Movie 3).

Several calcium sensitive dyes can be used with this approach depending on the nature of the experiment and the filters available in the microscope. Some of the most commonly used dyes, such as Fura-2 AM and the Fluo dyes, emit fluorescence in the green part of the spectrum. The Rhod-2 dye we used emits at a longer wavelength and, in our hands, was more reliable at these stages than the Fluo dyes. However, calcium transients in the mitochondria will also be visualized with Rhod-2. A calcium sensitive dye with dual emission peak like Indo-1 can be used for ratiometric measurements of intracellular calcium concentration. Dyes like Rhod-2 and the Fluo dyes can also be used for this propose if the embryos are loaded with a spectrally distinct calcium insensitive dye. Several new dyes have been developed to increase the fluorescence intensity, some examples include Quest Rhod-4 AM, Cal-520, and FluoForte.

The approach we described above can also be used to visualize other physiological events in mouse embryos that are closely related to calcium handling, such as membrane potential. For example, potentiometric dyes can be used separately or together with calcium sensitive dyes. Some of these dyes include fast-response dyes, like the ANEP dyes (Di-4-ANEPPS and Di-8-ANEPPS), and slow-response dyes like Oxonol VI. Another class of dyes that can be used are pH indicators, like pHrodo and Snarf-1. The approach can also be used to image genetically encoded calcium reporters and other fusion proteins in the developing cardiac tissue, without having to first remove the pericardium.

### Enhancing and quantifying contractile activity in image data

Contractile activity is a key biological parameter in developing cardiac tissue as its emergence represents a necessary transition toward a functioning heart. Myocyte contraction must always be preceded by a calcium transient: however it is not necessarily the case that an increase in cytoplasmic calcium leads to contraction in developing myocytes as the cell's contractile machinery may not be fully in place. We developed methods to measure contraction directly from image series data in order to compare cardiac developmental processes at the mechanical and ionic levels.

Contraction mapping has a long history in the cardiac sciences: motion can be used as a surrogate measure of electrical activation in tissue (Mines, 1914; Hwang et al., 2005) and, at the single cell level, image sequences can be analyzed to quantify sarcomere length and the dynamics of contraction/relaxation (Delbridge and Roos, 1997; Bazan et al., 2011). Measurement of cell motion in live embryos can give insights into cardiac mechanics and development (Forouhar et al., 2006).

Contraction can be difficult to quantify in raw image data because local motion transients deform tissue in an optically inconsistent way. Pixel intensity can either increase, decrease, or remain relatively constant with tissue motion, and the magnitude of the intensity change is only loosely correlated with the amplitude of tissue motion. This is in contrast to the use of intrinsic or exogenous probes which change in a monotonic and often linear fashion with their corresponding biological targets.

**Figure 4:**
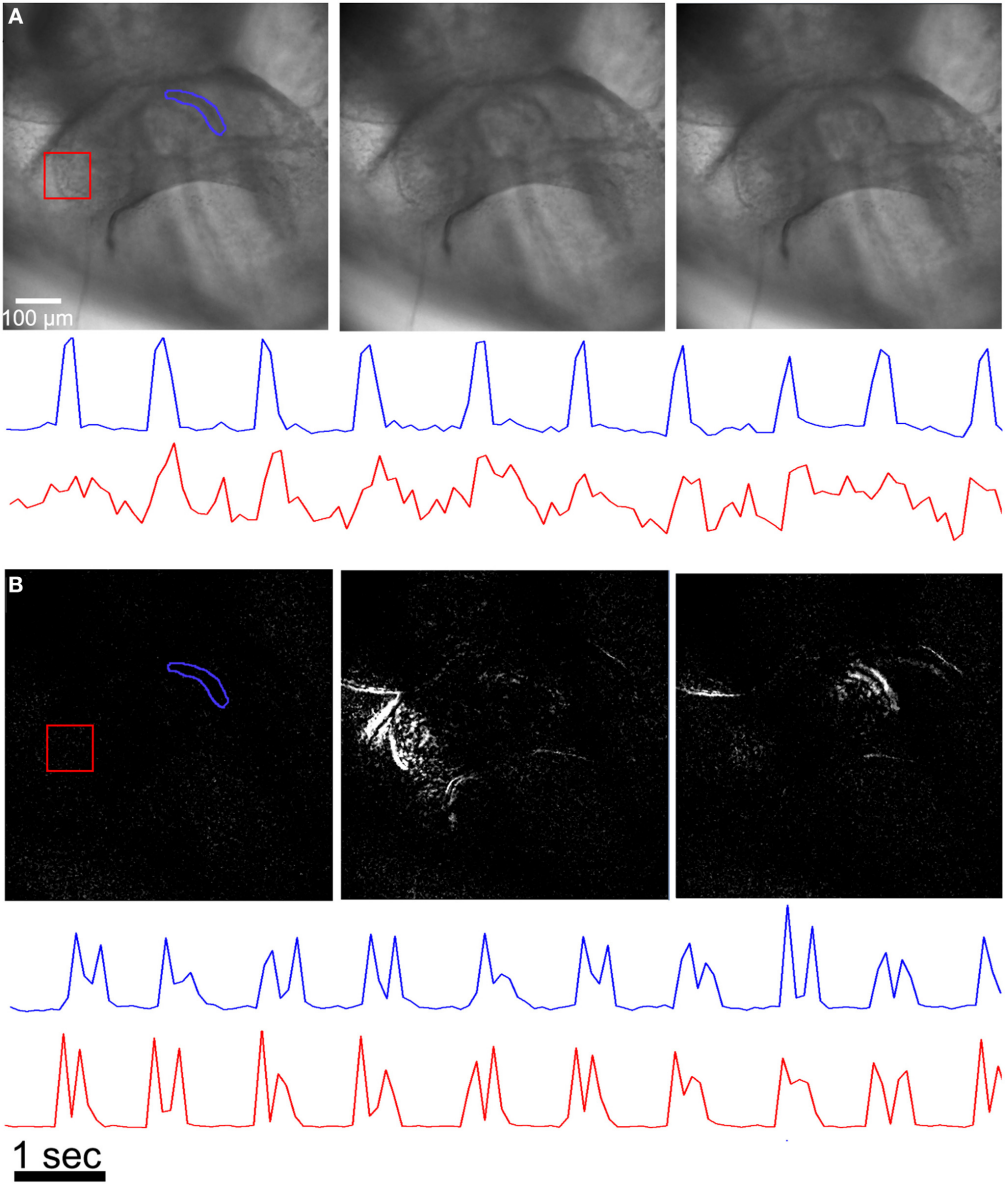
**Assessing contractility from image sequences. (A)** A ROI (region of interest) where the ROI is carefully chosen to surround a high contrast feature in the image (blue polygon and trace) can be used to assess periodicity when the intensity from all pixels within the region are plotted as a function of time. A ROI that contains both high and low contrast features yields an intensity vs. time plot that is difficult to interpret (red polygon and trace). **(B)** The image sequences are processed with an absolute difference image filter prior to plotting intensity vs. time plots from the same ROIs as in **(A)**. Here, both polygons give clear motion transients. The double spikes in the intensity vs. time plots are found in most regions, where the first spike is due to motion caused by tissue contraction, and the second spike is due to motion caused by tissue relaxation.

A simple method for quantifying contraction rate in an image series involves selecting a small region of interest (ROI) that contains pixels with a pronounced intensity difference compared to the surrounding tissue and tracking the intensity change in that ROI as a function of time. For example, if the ROI surrounds a relatively dark region made by a local high contrast ridge, then local tissue motion will lead to an increase in intensity as a fraction of the dark pixels are replaced by brighter pixels from the surrounding tissue (Figure 4A). This method can be used to accurately assess rhythmicity (inter-beat interval and its variance) at a few high contrast locations that have easily discernible motion within the sample.

**Figure 5:**
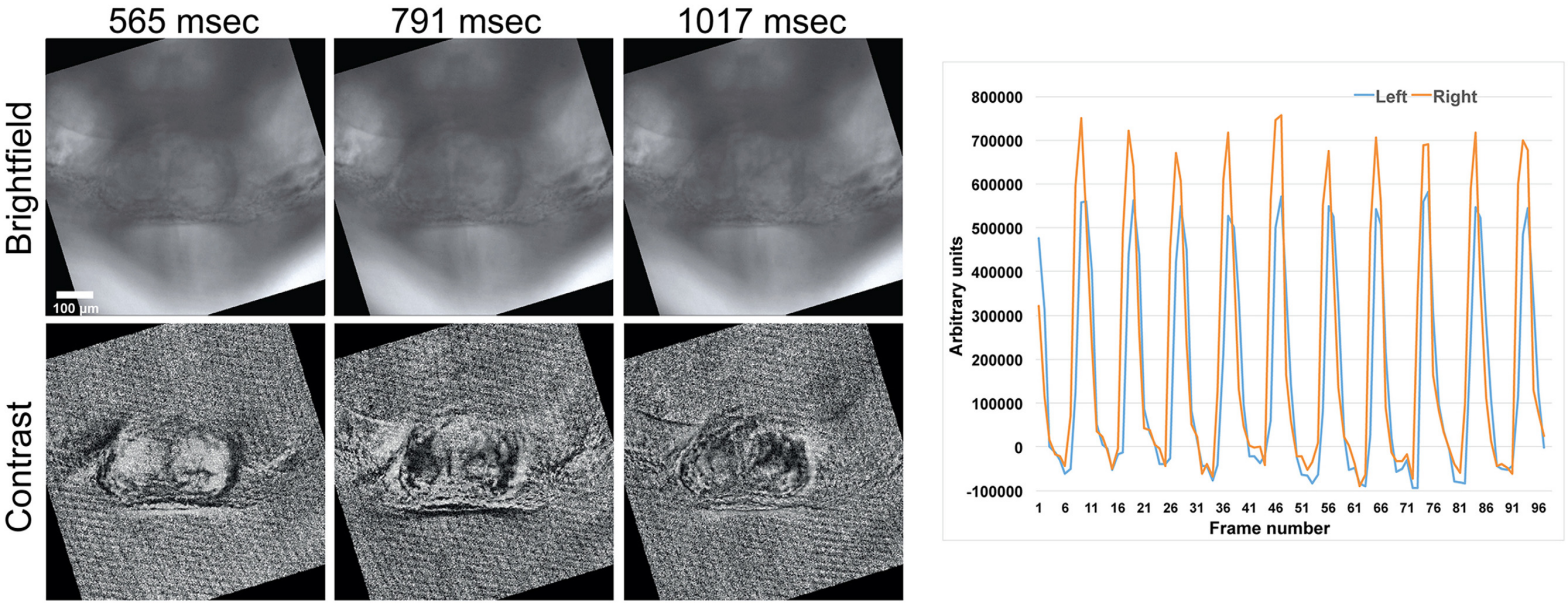
**A comparison between normal brightfield (top row, “Brightfield”) and temporally normalized images (bottom row, “Contrast”) obtained at different time points**. Large regions with uniform intensity (either black or white) within a single frame indicate parts of the tissue that have large motion transients. Intensity vs. time plots from ROIs within these regions are used to determine the timing of contraction. Here the initial site of contraction is on the right side of the heart (right panel). The scale bar represents 100 microns. Also see Supplementary Movie 4.

The location of moving regions within the sample can be visualized by applying a temporal normalization filter to the image sequence, which is similar to a standard histogram normalization filter, but here pixel scaling is applied in the time domain. A scale factor for each pixel location is found so that each pixels value varies over a constant range over the entire image sequence. Pixels from stationary regions will be scaled by spatially incoherent values set by inter-frame image noise that can be easily visually distinguished from moving regions which will be scaled in a spatially coherent fashion (Figure 5). Several ROIs for measuring rhythmicity at different locations within the field of view can then be chosen from a single normalized image without viewing the entire image sequence.

We implemented a variant of an absolute difference image filter in order to directly visualize spatial patterns of activation. Absolute difference image filters have been used in diverse motion tracking applications, ranging from visualizing cardiac activation waves in cell culture (Hwang et al., 2005) to tracking pedestrians in surveillance videos (Viola et al., 2005). The filter outputs the absolute value of each pixel's intensity relative to the same pixel N frames in the past, where N is a user controlled variable. Variable N is set so that the local intensity change is maximized, and in most cases this condition is met for any N greater than the action potential duration and less than the intrinsic periodicity (inter-beat interval) of the tissue. Similar results could be obtained by subtracting a single frame from all frames in the image sequence but a gradual drift in the sample location would degrade the measured intensity differences. Importantly, the absolute difference image filter allows us to measure motion for different regions in the sample without the need to carefully select a ROI (Figure 4B), which is advantageous when analysing a large number of image sequences where the embryo is in roughly the same position in the microscopes field of view.

### Detecting contractile activity by atomic force microscopy

Imaging modalities have limitations in sensitivity and resolution. In the case of brightfield microscopy, the contractile activity has to be of sufficient magnitude to displace pixels to an extent that they can reliably be detected at the resolution of the instrument. In the case of imaging calcium transients, the limitations are the sensitivity and speed of the detector and the responsiveness of the dye. In order to detect contractile activity at stages before we could reliably detect it visually, we used atomic force microscopy to detect the force generated by the expansion and contraction of embryonic tissues as a result of the sub-micron physical displacement caused by contraction.

Atomic force microscopes (AFM) can be used to detect nano-newton scale forces using a flexible cantilever to probe the sample. Very small deflections of the cantilever are detected by measuring the displacement of a laser beam reflected off the top of the cantilever. The deflection of the cantilever can then be used to model the physical characteristics of the sample, such as its Young's modulus (Franze, 2011).

**Figure 6:**
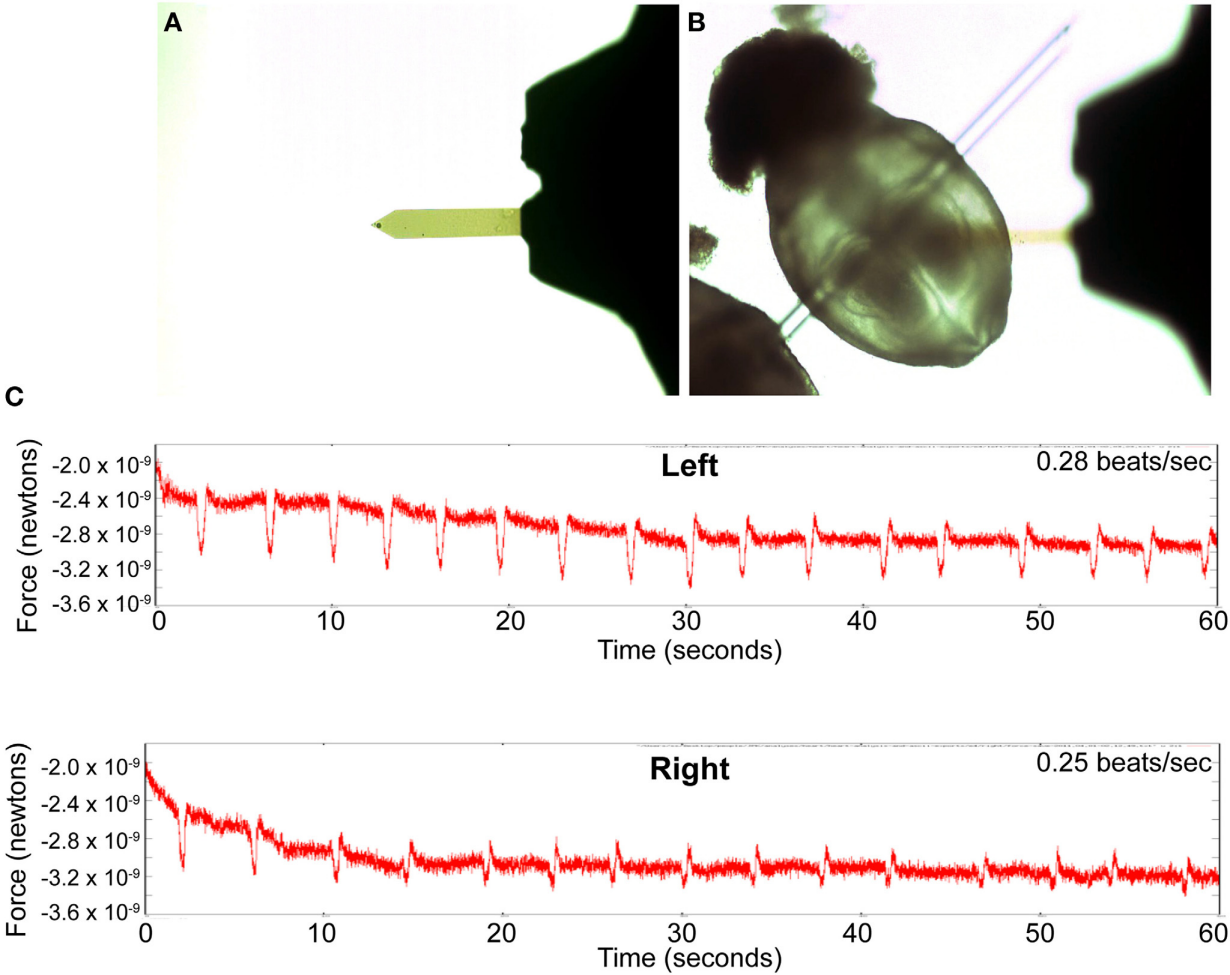
**Detection of contractile activity using atomic force microscopy. (A)** The AFM cantilever with an 11 micron latex bead affixed to the tip. **(B)** Brightfield image of an embryo immobilized by inserting a hand pulled glass filament through the yolk sac. The cantilever of the AFM can be seen out of focus above the embryo. **(C)** Plots of the rhythmic changes in force measurements on the right and left sides of the cardiac crescent, reflecting the contractile activity in these regions. The values on the y-axis represent relative changes in the force spectrum measured by the AFM probe on the surface of the embryo and are not direct measurements of the contractile force exerted by underlying embryonic cardiomyocytes.

The AFM instrument sits on the stage of a standard inverted compound microscope and the probe is lowered onto the sample while it is monitored optically from below. As a result, we could not use the embryo immobilization approach we used for light microscopy, which has arches of vacuum grease on top of the embryo. Therefore, to immobilize the embryo, we used a short fragment of a fine hand-drawn glass capillary inserted through the yolk sac (Figure 6B). The weight of the capillary is sufficient to hold the embryo firmly on the floor of the culture dish. We inserted the glass capillary not in the center of the embryo but somewhat superficially, toward the caudal region, so that the glass rod was already near the floor of the dish and did not tear through the yolk sac as it settled to the bottom of the dish. We were able to easily control the orientation of the embryo in this way. This approach however results in the puncturing of the yolk sac and therefore is not suitable for long term culture. It is however adequate for acute short-term experiments to measure contraction at a single stage in development.

To determine if we can detect contractions in the developing cardiac primordium before we can visually see it, we dissected embryos in M2 medium and immobilized them in a dish of M2 as described above. We prepared the AFM cantilever with an 11 micron bead to act as our probe (Figure 6A). The bead allowed us to take measurements from the embryonic tissue without damaging it. We next probed the right and left sides of the developing cardiac primordium by lowering the probe onto the appropriate locations of the embryo while optically monitoring it with the compound microscope. We observed a rhythmic variation in the force detected by the AFM probe when placed on the left and right sides of the developing heart primordium (Figure 6C). We were unable to visually detect any contractile activity with the compound microscope in these samples, indicating that the AFM was detecting contractile activity that was of too low a magnitude to be visible.

### Conflict of interest statement

The authors declare that the research was conducted in the absence of any commercial or financial relationships that could be construed as a potential conflict of interest.

## Supplementary material

Supplementary Figure 1 **Schematic illustrating the setup of the glass bottom dish used to image embryos. (A)** Embryos were either immobilized under arches of vacuum grease or **(B)** between two rails of vacuum grease with transverse “rungs.”

Supplementary Movie 1 **Time-lapse movie of brightfield, GFP, and merged channels of an E7.75 embryo**. The egg cylinder is orientated with the distal end toward the bottom, the proximal end toward the top and the forming rostral aspects facing the viewer. Hex-GFP labels cells of the endoderm. The scale bar represents 100 microns and the time in culture is shown at the bottom as hours:minutes. Also see Figure 1.

Supplementary Movie 2 **Time-lapse movies taken at various stages in the development of an E7.75 embryo, developing from the cardiac crescent to linear heart tube stages**. The time above each image is measured from the start of culture. Such a series of time-lapse movies allows one to clearly visualize the development of contractile activity in the forming heart. The scale bar represents 100 microns and applies to all the sequences. The time is shown to the right in milliseconds and applies for all the sequence. Also see Figure 2.

Supplementary Movie 3 **Time-lapse sequence of an E8.0 linear heart tube imaged for Ca^2+^ transients by confocal microscopy**. The transients were visualized by staining with Rhod-2 and are shown with a spectrum LUT with bluer colors being dimmer and redder colors being brighter. The scale bar represents 100 microns. Also see Figure 3.

Supplementary Movie 4 **Time-lapse movie of a linear heart tube stage embryo showing a comparison between the captured brightfield and contrast enhanced image data**. The scale bar represents 100 microns. Also see Figure 5.
