# Supplementary figures and images for: Detecting cardiac contractile activity in the early mouse embryo using multiple modalities
Source: Front Physiol. 2015 Jan 7;5:508. doi: 10.3389/fphys.2014.00508 (PMC4285868; doi:10.3389/fphys.2014.00508)

A

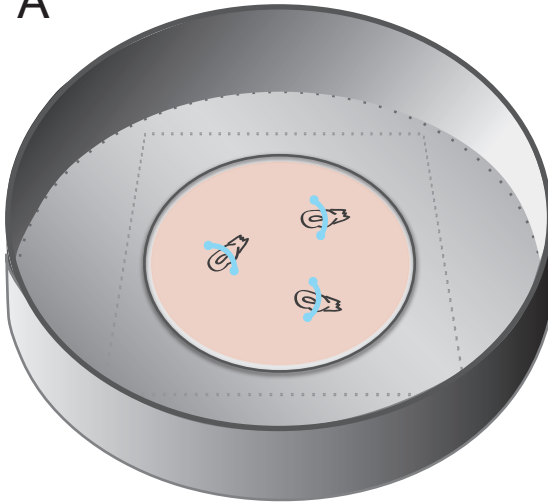

B

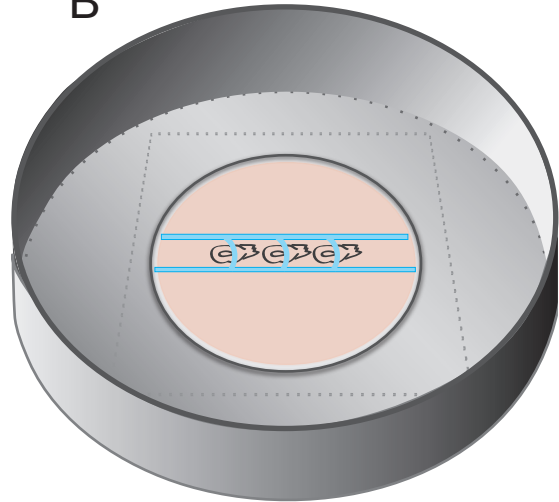

- 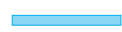 Vacuum grease
- 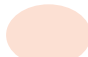 Culture medium
- 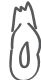 Embryo
- 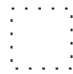 Glass bottom of dish

Supplement: Supplementary Figure 1 — Schematic illustrating the setup of the glass bottom dish used to image embryos. (A) Embryos were either immobilized under arches of vacuum grease or (B) between two rails of vacuum grease with transverse “rungs.” [file Image1.PDF]
